# Estimating the direct costs of oral cancer in Spain: a retrospective hospital data analysis

**DOI:** 10.4317/medoral.25847

**Published:** 2023-06-18

**Authors:** Marta Porta-Vázquez, José Luis López-Cedrún, Jacinto Fernández-Sanromán, Eliane García-Mato, Pedro Diz-Dios, Márcio Diniz-Freitas

**Affiliations:** 1 DDS, Medical-Surgical Dentistry Research Group (OMEQUI), Health Research Institute of Santiago de Compostela (IDIS), University of Santiago de Compostela (USC), Santiago de Compostela5/5/2023, Spain; 2 MD, PhD, Department of Oral and Maxillofacial Surgery, A CorunÞa University Hospital Complex (CHUAC), A CorunÞa, Spain; 3 MD, PhD, Department of Oral and Maxillofacial Surgery, Hospital Rivera POVISA, Vigo, Spain; 4 MD, DDS, PhD, Medical-Surgical Dentistry Research Group (OMEQUI), Health Research Institute of Santiago de Compostela (IDIS), University of Santiago de Compostela (USC), Santiago de Compostela, Spain; 5 DDS, PhD, Medical-Surgical Dentistry Research Group (OMEQUI), Health Research Institute of Santiago de Compostela (IDIS), University of Santiago de Compostela (USC), Santiago de Compostela, Spain

## Abstract

**Background:**

Studies on the costs incurred from cancer in Spain are scarce and have focused on the most prevalent types such as colorectal, breast, and lung cancer. The aim of this study was to calculate the direct costs associated with the diagnostic, treatment and follow-up procedures for oral cancer in Spain.

**Material and Methods:**

Applying a bottom-up approach, we retrospectively analyzed the medical records of a cohort of 200 patients with oral cancer (C00-C10), diagnosed and treated in Spain between 2015 and 2017. For each patient, we collected their age, sex, degree of medical impairment (American Society of Anesthesiologists [ASA] classification), tumor extent (TNM classification), relapses and survival during the first 2 years of follow-up. The final calculation of the costs is expressed in absolute values in euros as the percentage of the gross domestic product per capita and in international dollars (I$).

**Results:**

The total cost per patient rose to €16,620 (IQR, €13,726; I$11,634), and the total direct cost at the national level was €136,084,560 (I$95,259,192). The mean cost for oral cancer represented 65.1% of the gross domestic product per capita. The costs for the diagnostic and therapeutic procedures were determined by the ASA grade, tumor size, lymph node infiltration and presence of metastases.

**Conclusions:**

The direct costs for oral cancer are considerable compared with other types of cancer. In terms of gross domestic product, the costs were similar to those of countries neighboring Spain, such as Italy and Greece. The main determinants of this economic burden were the patient’s degree of medical impairment and tumor extent.

** Key words:**Mouth neoplasms, cost analysis, healthcare costs, cohort study.

## Introduction

According to estimates by the Global Cancer Observatory, 529,708 cases of lip, oral cavity, salivary gland and oropharyngeal cancer were diagnosed worldwide in 2020, representing 2.8% of all cancers (1). Although there has been a modest reduction in the number of patients with lip and oral cavity cancer in Europe in the past 30 years, the incidence rate is still 5.3 new cases per 100,000 inhabitants, and the high premature mortality of these patients continues to be a public health problem (2).

Studies on the costs of treating oral and oropharyngeal cancer have special importance despite its relatively low incidence compared with other types of cancer, given that the former is often detected in advanced stages, which demands more complex and expensive treatments (3). A systematic review was published in 2014 on the economic burden of head and neck cancer, which included articles published between 2003 and 2013 (4). The authors emphasized that this type of study provided valuable economic information for payers, providers and patients; however, most of the selected studies were performed in the United States; the authors therefore stressed the need for conducting this type of study in Europe (4). A new systematic review was recently published on the costs of oral cancer, which included articles published between 2001 and 2021 (5). The selected articles were based on information from patients from 15 different countries, 7 of them European (Italy, The Netherlands, Greece, France and England) (5), but did not include Spain.

According to data provided by the Spanish Society of Medical Oncology (https://seom.org/images/Cifras_del_cancer_en_Espnaha_2021.pdf, in Spanish), it is estimated that 8188 cases of oral cavity and pharyngeal cancer were diagnosed in Spain in 2021, placing this type of cancer in eighth place among all types diagnosed in Spain, only behind colorectal, prostate, breast, lung, bladder/urinary, non-Hodgkin’s lymphoma and pancreatic cancer. The studies on the costs incurred by cancer in Spain are scarce and have focused on the most prevalent tumors such as colorectal, breast and lung cancer. The aim of this study was to calculate the direct costs associated with the diagnostic, treatment and follow-up procedures for oral cancer in Spain.

## Material and Methods

- Study design

We retrospectively analyzed the medical records of a cohort of 200 patients with oral cancer, diagnosed and treated in the maxillofacial surgery departments of 2 hospitals of the Autonomous Community of Galicia (Spain) between 2015 and 2017 (University Hospital Complex of A CorunÞa and Hospital Ribera Povisa of Vigo).

All of the selected patients had squamous cell carcinoma categorized, based on its location, as C00-C10 according to the International Classification of Disease [ICD-10] (https://www.who.int/standards/classifications/classification-of-diseases). We included lip (C00), oral cavity (C01-C06), salivary gland (C07-C08) and oropharyngeal cancer (C09, C10), which we classified under the common epigraph of “oral cancer” (OC).

In each case, we collected the patients’ demographic data (age and sex), their degree of medical impairment according to the American Society of Anesthesiologists (https://www.asahq.org/standards-and-guidelines/asa-physical-status-classification-system), tumor-related information according to the American Joint Committee on Cancer TNM Staging System (https://www.facs.org/quality-programs/cancer-programs/american-joint-committee-on-cancer/cancer-staging-systems/) and relapses and survival during the first 2 years of follow-up.

Permission to access the patient files was provided by the Galician Health Service and by the Management of Hospital Ribera POVISA. Ethical approval for the study was provided by the Galician Research Ethics Committee (Reference 2014/604).

- Cost analysis

The cost analysis was based on individual data (bottom-up approach), obtained by multiplying each procedure’s unit costs by the number of times the procedure was performed. We quantified the direct costs generated by each patient from their OC diagnosis to the end of treatment of the primary tumor. Additionally, we quantified the expenses resulting from the incidences recorded during the follow-up of the disease or, if necessary, until the patient’s death as a result of the cancer during this period. The costs were calculated by applying the current official rates for each phase of the process, rates published by the Galician Health Service in 2014 (https://www.xunta.gal/dog/Publicados/2014/20140521/AnuncioC3K1-140514-0001_es.html, in Spanish).

The final cost calculation is expressed in euros as the percentage of the gross domestic product (GDP) per capita and in international dollars (I$) by purchasing power parity (PPP) (https://data.worldbank.org/indicator/PA.NUS.PPP).

The following diagnostic procedures were considered: imaging techniques (orthopantomography, ultrasonography, chest and abdomen radiography, Head and Neck, chest and abdomen computed tomography [CT], magnetic resonance imaging [MRI]), laboratory studies (blood test including complete blood count, biochemical profile and coagulation study, blood cultures, fine needle aspiration, biopsy sampling and pathology study), interconsultations with physicians of various specialties and preoperative assessment (chest radiography, blood test, electrocardiogram and anesthesiologist assessment).

The therapeutic procedures whose costs were quantified were as follows: surgery under local anesthesia, surgery under general anesthesia, neck dissection, tracheotomy, plate osteosynthesis, duration of hospitalization (surgery-related), radiation therapy sessions (regardless of the technique and voltage applied) and chemotherapy cycles.

- Statistical methodology

The statistical analysis of the results was performed using the free software R (R Development Core Team 2017, Vienna, Austria). To analyze the difference in costs (treatment and diagnostic) according to the patient’s demographic variables and degree of medical impairment, we conducted a normality analysis (Shapiro-Wilk test). The influence of sex was analyzed using the Wilcoxon test, while age and ASA score were analyzed by applying the Kruskal-Wallis test. We also employed the Kruskal-Wallis test to study the variation in the costs according to the tumor’s characteristics (TNM).

## Results

- Patient and tumor characteristics

Of the 200 patients who comprised the study group, 131 were men (65.5%) and 69 were women (34.5%). The mean age at the time of diagnosis was 65.0±13.8 years (range, 26-90 years). The degree of medical impairment corresponded to ASA level I in 14% of the cases, ASA II in 46%, ASA III in 39.5% and ASA IV in 0.5%.

Ninety percent of the tumors were located in the oral cavity (C01-C06), 4.5% in the oropharynx (C09-C10), 3.5% in the salivary glands (C07-C08) and 2% in the lips (C00). In terms of tumor size, 35% of the patients had T1 tumors, 41% had T2, 17% had T3 and 7% had T4. With regard to lymph node infiltration, 61% of the patients were categorized as N0, 24% as N1, 11% as N2, 3% as N3 and 1% as N4. Some 11.5% of the patients had extraoral metastases at the time of diagnosis, and 16% presented relapses during the follow-up.

- Diagnostic procedures

The total cost of the diagnostic procedures rose to €800,668, with a median of €3602 (IQR, €2162) per patient. The variables that determined this cost were the patient’s degree of medical impairment (*p*= 0.037), tumor size (*p*< 0.001), the presence of infiltrated lymph nodes (*p*<0.001) and the presence of metastases (*p*< 0.001) (Table 1).

**Table 1:**
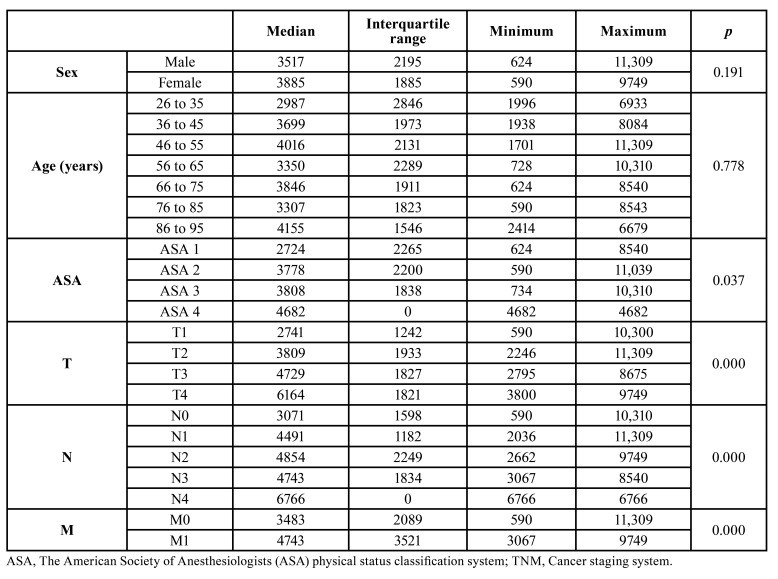
Costs (in euros) for the diagnostic procedures in patients with oral cancer according to the patient’s characteristics and tumor extent.

The most widely used imaging techniques were Head and Neck CT (*n*=362), chest radiography (*n*=332) and orthopantomography (*n*=208). The total cost of the imaging tests was €230,873, especially due to Head and Neck CT (€136,604) and MRI (€41,477). Table 2 details the tumor extent variables that determined the cost of each of the imaging tests conducted for diagnostic purposes. Head and Neck CT was the only procedure determined by the 3 variables that constitute TNM staging (*p*< 0.001, *p*= 0.002 and *p*= 0.001, respectively).

The most widely used additional diagnostic tests were blood tests (complete blood count, biochemical profile and coagulation study) (*n*=324), pathology studies (*n*=293) and biopsy sampling (*n*=284). The total cost of the additional diagnostic tests was €283,573, especially due to blood tests (€136,835) and pathology studies (€110,657). Pathology studies and biopsy sampling were the only procedures determined by the 3 variables that constitute TNM staging (*p*< 0.001 for the 3 variables) (Table 2).

**Table 2:**
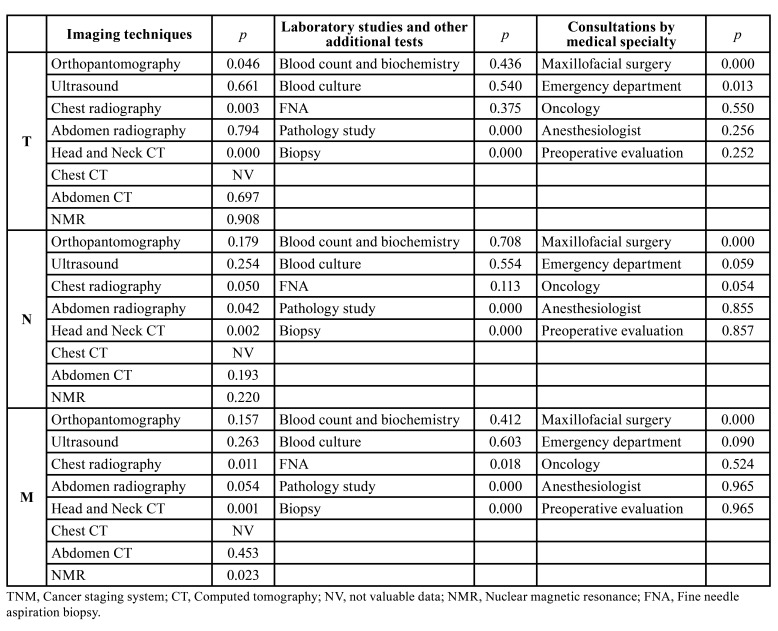
Significance levels of the influence exerted by the tumor extent variables on the costs of the diagnostic procedures in patients with oral cancer.

The most common consultations by medical specialty were maxillofacial surgery (*n*=2550) and oncology (*n*=796). The total cost of these consultations was €286,222, especially due to preoperative assessments (€140,445) and maxillofacial surgery consultations (€88,281). The maxillofacial surgery consultations were the only ones determined by the 3 variables that constitute TNM staging (*p*< 0.001 for the 3 variables), and emergency department consultations were conditioned by tumor size (*p*= 0.013) (Table 2).

- Therapeutic procedures

The total cost of the therapeutic procedures rose to €2,563,328, with a median of €11,291 (IQR, €11,822) per patient. The variables that determined this cost were the patient’s degree of medical impairment (*p*< 0.001), tumor size (*p*< 0.001), the presence of infiltrated lymph nodes (*p*< 0.001) and the presence of metastases (*p*< 0.012) (Table 3).

**Table 3:**
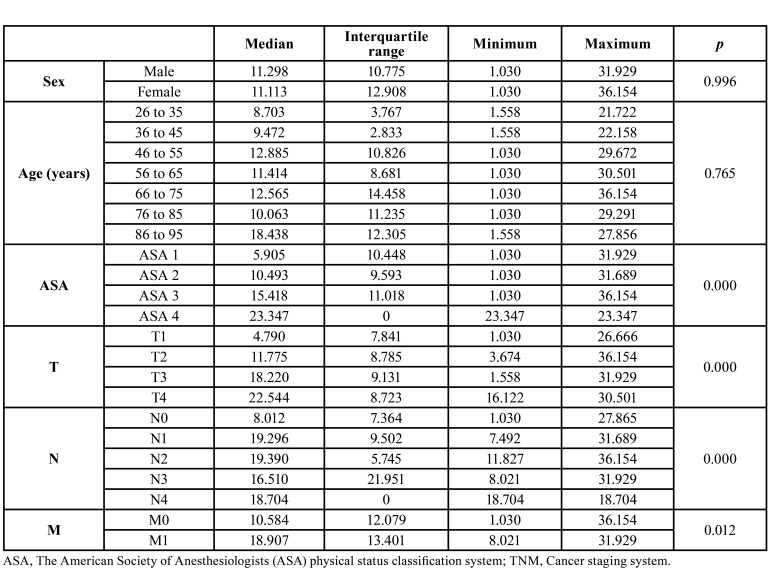
Costs (in euros) for the therapeutic procedures in patients with oral cancer according to the patient’s characteristics and tumor extent.

The most frequent surgical interventions were tumor resection under general anesthesia (*n*=193) and Head and Neck lymph node dissection (*n*=125). The total cost of the surgical interventions was €2,155,558, of which €1,069,095 corresponded to strictly surgical procedures and €1,086,463 covering the costs of hospitalization (*n*=2054). The total cost of the radiation therapy was €304,851, while that of chemotherapy was €102,919. Table 4 details the tumor extent variables that determined the cost of each of the therapeutic procedures; the 3 variables that constituted the TNM staging determined the surgical hospitalization duration (*p*< 0.001, *p*< 0.001 and *p*< 0.001, respectively) and the radiation therapy cycles (*p*= 0.006, *p*< 0.001 and *p*= 0.040, respectively).

- Follow-up

The total direct cost during follow-up was €403,793, of which €187,915 corresponded to diagnostic procedures and €215,878 to therapeutic procedures, which represents an approximate increase of €1397 per patient.

The total cost for imaging tests was €80,659, especially due to cervical CT (*n*=177; €136,604). The most widely used additional diagnostic tests were blood tests (*n*=81), pathology studies (*n*=42) and biopsy sampling (*n*=35). The total cost for the additional diagnostic tests was €54,197, especially due to blood tests (€34,209). The most common consultations by medical specialty were maxillofacial surgery (*n*=975) and oncology (*n*=154). The total cost for these consultations was €53,059, especially due to the maxillofacial surgery consultations (€33,754).

The most frequent surgical interventions were tumor resections under general anesthesia (*n*=19) and local anesthesia (*n*=13). The total cost for the surgical interventions was €186,686, of which €97,823 corresponded to strictly surgical procedures and €88,863 covering the costs of hospitalization (*n*=168). The total cost for radiation therapy was €21,775, while that for chemotherapy was €7417.

- Total cost and estimate at the national level

The median joint cost for the diagnostic and therapeutic procedures was €15,223 (IQR, €13,594) per patient. Assuming that the number of cases of oral cancer diagnosed in Spain in 2021 was 8188 and that their percentage distribution based on size (T1-T4) was similar to that of the present series, applying the value of the cost per patient that we determined, the total estimated annual direct cost for OC in Spain would be €124,645,924. If we also consider the additional costs of treating the primary tumor during the 2-year follow-up, the total cost per patient rises to €16,620 (IQR, €13,726), which is equivalent to I$11,634, and the total direct costs calculated at the national level would be €136,084,560, which equivalent to I$95,259,192.

**Table 4:**
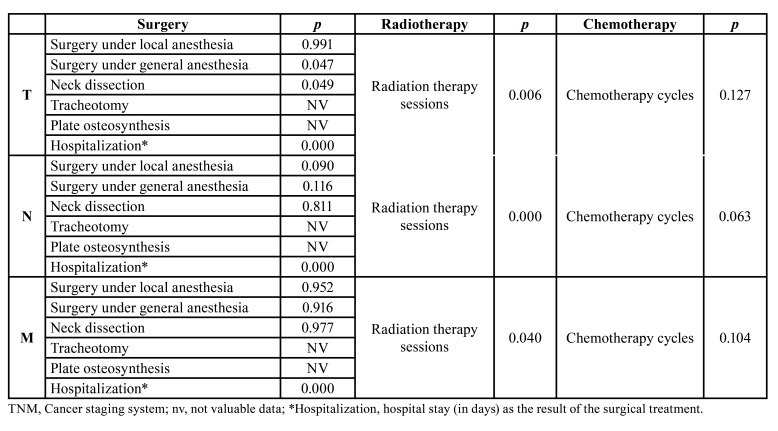
Significance levels of the influence exerted by the tumor extent variables on the costs of the therapeutic procedures in patients with oral cancer.

The GDP per capita of Spain in 2021 was €25,500; therefore, the mean cost of OC represents 59.6% of the per capita GDP. If we include the expenses of the first 2 years of follow-up, the percentage increases to 65.1%.

## Discussion

To our knowledge, this is the first report on the costs that squamous cell carcinoma incurs in Spain. The main finding of our study is that the direct estimated costs for the diagnosis, treatment and follow-up of patients with OC (C00-C10) represent a considerable economic burden, which is fundamentally determined by the tumor extent (TNM).

For 2015, the calculated total cost of cancer in Spain was €5458 million, of which €4818 million corresponded to direct costs (6). Applying the results of the present study, the expenses attribuTable to OC represent approximately 2.5% of the total cost. This percentage takes on greater relevance if we consider that, in Spain, the direct costs of OC per patient are higher than those generated in 3 years by colorectal, breast, prostate or lung tumors in stages I to III (7). This result coincides with those previously published in the United States, where the costs of OC (C01-C10) have been reported to be the highest of all cancers (8).

It is very difficult to compare the estimated costs in Spain with those of other countries, given that most of these studies jointly analyzed all head and neck cancers (5). Additionally, a number of these publications are 10 to 20 years old, which requires a recalculation of their estimates applying inflation rates to the present time (DatosMundial.com, eglitis-media, Oldenburg, Germany). Most of the authors who broke down the financial analysis of OC by tumor location agree that lip cancer incurs lower costs than oral cavity cancer, which in turn has lower costs than that of oropharyngeal cancer (9,10); however, this order is not shared by other researchers (3). Although this study did not break down the costs by tumor location, we can assume that the mean costs of all cases of OC represent mainly those of oral cavity tumors (C01-C06), given that these constitute 90% of all tumors included in the present series. Compared with countries neighboring Spain, the direct cost of OC in Spain in absolute values was higher than that in Greece (€8450; applying the inflation rate ~€11,398) (11), similar to that in Italy (€19,028) (10) and obviously lower than that of The Netherlands (€25,425; applying the inflation rate ~€36,422) (12) and UK (£25.311; applying the inflation rate ~€35,052) (13).

In terms of GDP per capita, the direct costs of oral cavity cancer in Spain were similar to those in Greece (65%) (11) and Italy (64.9%) (10) and lower than those in the UK (79.8%) (13), the Netherlands (79.8%) (12) and in general to the mean cost in Western countries (75%) (5). Despite the recommendations of the World Health Organization (14), we found only one study on OC costs in which the results were expressed in international dollars, the authors of which calculated that the cost per process in Brazil was I$23,924,3 duplicating the estimates of the direct costs in Spain.

The patient’s sex did not affect the direct costs of OC, coinciding with the results of other published series (10,15). However, other studies conducted in France and China have indicated that the direct costs of head and neck tumors were higher in men than in women, arguing that the cancer is typically diagnosed in earlier stages in women (9,16).

Coinciding with previous publications, the patient’s age did not affect the costs of OC (16). In contrast, a number of authors have suggested that the costs of head and neck cancer decrease as the patient’s age increases (9,10).

The ASA classification, a universally applied and easy-to-obtain assessment of the patient’s health status, is positively related to the costs of OC. We found only one reference in the literature in this regard, which reported no significant relationship between ASA level and the costs of oral cavity cancer, although this small series (*n*=73) published in 1998 included only 3 patients with ASA I and 1 with ASA IV (17). However, the ASA classification has been the most widely used comorbidity index to assess the results of the surgical treatment of head and neck tumors (18), and a positive correlation has been confirmed between the presence of comorbidities and the costs of head and neck tumors (19).

Coinciding with the results of our study, there is unanimous agreement in the literature regarding the influence of tumor extent on the direct costs of OC, such that the costs increase as the tumor staging increases (10,11,20-23).

This study has a number of limitations that need to be considered when interpreting the results and comparing them with other published studies. We assessed tumors from various locations, occasionally not well delimited, which are difficult to compare with other studies in which a non-standardized terminology was applied (5). We did not consider known risk factors, and it has been shown that tobacco use and the presence in the tumor of the human papillomavirus affect the costs of head and neck cancer (16,24). As with most studies on the economic burden that OC represents, this study assessed the direct healthcare costs (3,13,21,23,25), although they were underestimated because we did not include the expenses resulting from deferred reconstructive surgery (after the 2-year follow-up) or those of prosthetic rehabilitation, which obviously significantly increase the total cost. Unlike other authors (3,13,16,21,23), we did not quantify the consumption of non-healthcare resources such as transport, home expenses, relocation, property losses and informal care (26). We also did not assess the indirect costs resulting from work absenteeism, which represent between 5% and 15% in relation to the direct costs (9,25,27). Lastly, the indirect expenses also include those resulting from early mortality, which are the most relevant (23), can significantly exceed the direct costs (25) and can generate significant economic losses, especially in countries with limited resources (28), although OC mortality has significantly declined in Spain in recent years (29).

As suggested by Mignogna *et al*. 20 years ago, strategies need to be developed for preventing and diagnosing OC early, with the objective of increasing patient survival, improving their quality of life, and reducing the enormous consumption of healthcare resources that OC incurs (30). In an ideal situation in which all tumors of the present study are diagnosed in stage T1, the total cost of the initial diagnostic and treatment procedures would be €65,594,068, which involves a reduction in the direct costs at the national level of 47.4% (€59,051,856). In a more realistic situation in which 35% of tumors are T1 (such as in the present series) and all the remaining are diagnosed in stage T2, the initial total cost would be €106,163,674, which would lead to a reduction in direct costs at the national level of 14.9% (€18,482,250).

## Conclusions

Although they have been underestimated, the direct costs of OC calculated in this study were considerable and greater than those of other types of cancer. In terms of GDP, the costs were similar to those of countries neighboring Spain, such as Italy and Greece, although lower than those of other Western countries. The main determinants of this economic burden were the patient’s degree of medical impairment and tumor extent. Accordingly, the best method for reducing the direct costs of OC is probably continuing to implement initiatives to eliminate the known risk factors and promote early tumor diagnosis.
